# Dissecting discordance of mitochondrial and nuclear phylogenetic trees in insects

**DOI:** 10.1007/s44297-025-00062-3

**Published:** 2025-12-12

**Authors:** Xianfeng Mi, Guo-Zheng Ou, Yixiao Zhu, Xing-Xing Shen

**Affiliations:** 1 Zhejiang Key Laboratory of Biology and Ecological Regulation of Crop Pathogens and Insects, Institute of Insect Sciences, College of Agriculture and Biotechnology, Zhejiang University, Hangzhou, China; 2 Yazhou Bay Science and Technology City, Hainan Institute, Zhejiang University, Sanya, China

**Keywords:** Phylogenetics, Mito-nuclear phylogenetic discordance, Insect genomics, GC content

## Abstract

The mitochondrial and nuclear genomes present distinct inheritance and genomic structural characteristics. Although both genomes have been widely used for phylogenetic tree reconstruction, topological discordances between trees inferred from mitochondrial and nuclear genomes—referred to as mito-nuclear phylogenetic discordance—have been widely reported. In this study, we analyzed the mitochondrial and nuclear genomic data from 472 insects and systematically investigated the impact of gene properties on the mito-nuclear phylogenetic tree discordance. Our analyses revealed that this discordance was not attributed to most examined factors, such as gene length and gene tree resolution, with the exception of guanine-cytosine content (GC%). We found that the mitochondrial genes exhibited significantly lower GC% compared to the nuclear genes. Further analyses revealed that the mito-nuclear phylogenetic discordance can be reduced by sampling mito-like nuclear genes that possess a similar GC content to that of mitochondrial genes. Moreover, we found that within nuclear genomes, low-GC nuclear genes are more topologically similar to each other and exhibit different biological functions than high-GC nuclear genes. These findings enhance our understanding of the mito-nuclear phylogenetic tree discordance in insects.

**Supplementary Information:**

The online version contains supplementary material available at 10.1007/s44297-025-00062-3.

## Introduction

Phylogenetic trees reconstructed from mitochondrial and nuclear genes often exhibit incongruent topologies, a phenomenon widely referred to as mito-nuclear phylogenetic discordance. Such incongruence has been extensively reported across a broad range of lineages, including vertebrates (birds, fishes, turtles, mammals), arthropods (insects, arachnids), fungi, protozoans, and cnidarians [1–13]. This widespread discordance hampers efforts to reconstruct accurate evolutionary relationships and to interpret patterns of important trait evolution.

The structure, inheritance, and evolutionary rate of mitochondrial and nuclear genomes differ fundamentally [14–17]. The mitochondrial genome is haploid, maternally inherited, compact, and non-recombining, whereas the nuclear genome is diploid, biparentally inherited, and undergoes recombination every generation. Moreover, mitochondrial genomes typically evolve faster and contain fewer genes. Although both genomes harbor valuable phylogenetic signals, these intrinsic differences may result in topological conflicts between mitochondrial and nuclear phylogenies.

Insecta, the most species-rich class of arthropods [20, 21], provides an ideal model for investigating the causes of mito-nuclear phylogenetic discordance. As a highly diverse group encompassing nearly all major arthropod lineages, insects display remarkable variation in genomic architecture, base composition, and evolutionary dynamics [22, 23]. Recently, Tao et al. reported a large-scale dataset comprising 472 insect species from 19 orders [24], offering an opportunity to examine how gene heterogeneity contributes to mito-nuclear topological discordance at a phylogenomic scale.

Previous studies have proposed various biological and methodological explanations for mito-nuclear phylogenetic discordance among some insect lineages, such as butterflies, beetles, and caddisflies [1, 12, 18, 19]. However, a broader investigation of mito-nuclear phylogenetic discordance across different insect orders is lacking, and the potential influence of genomic properties—such as the GC content, alignment length, substitutional saturation, and other compositional features—has not been systematically evaluated.

In this study, we leveraged a large-scale insect dataset to dissect mito-nuclear phylogenetic discordance from the perspective of genomic heterogeneity. Note that our aim is to elucidate the cause of mito-nuclear discordance rather than to determine which genome provides the true or better phylogenetic tree. Specifically, we examined whether differences in nine gene properties, such as gene alignment length and GC content, could explain mito-nuclear phylogenetic discordance; and we explore whether the mito-like nuclear genes could reduce the observed phylogenetic discordance.

## Results

### Incongruence between mitochondrial and nuclear phylogenies

**Fig. 1:**
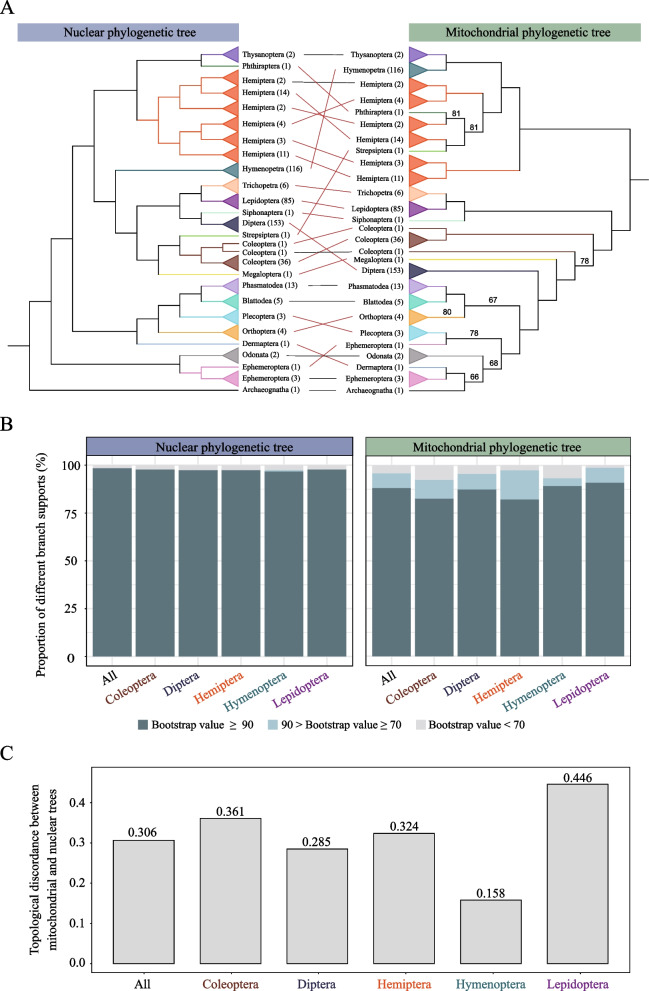
Discordance between the nuclear and mitochondrial phylogenies of 472 insects. **A** Comparison of the concatenated maximum likelihood (ML) phylogenies reconstructed from 1,367 single-copy nuclear protein-coding genes (left) and 13 mitochondrial protein-coding genes (right). Branch colors represent distinct insect orders, and numbers in parentheses represent species counts per clade. The red dashed lines indicate conflicting associations, whereas the black lines indicate congruent associations. Nodes with ultrafast bootstrap values below 90 are shown. **B** Proportions of different branch supports for all insects and five major orders in the nuclear and mitochondrial phylogenies. **C** Topological discordance between the mitochondrial and nuclear phylogenies for all the insects and the five major orders. The topological discordance between the mitochondrial and nuclear phylogenies was measured as the normalized Robinson–Foulds (nRF) distance. The numbers of species analyzed in All, Coleoptera, Diptera, Hemiptera, Hymenoptera, and Lepidoptera are 472, 38, 153, 36, 116, and 85, respectively

To assess the extent of mito-nuclear phylogenetic discordance across insects, we first reconstructed two concatenation-based maximum likelihood (ML) phylogenies for 472 insect species, using 1,367 single-copy nuclear genes and 13 mitochondrial protein-coding genes (mtPCGs) (Fig. 1A). Overall, both the nuclear and the mitochondrial phylogenies presented high branch support (Fig. 1B). Specifically, 98.30% of nodes in the nuclear phylogeny and 88.11% in the mitochondrial phylogeny presented bootstrap values ≥ 90%, indicating high resolution in both topologies. Within the five largest insect orders-Coleoptera, Diptera, Hemiptera, Hymenoptera, and Lepidoptera-the proportion of highly supported nodes (bootstrap value ≥ 90%) remained above 95% in the nuclear trees and above 80% in the mitochondrial trees (Fig. 1B). These results show that both inferred mitochondrial and nuclear phylogenies are generally well- supported.

To visualize topological differences between the two datasets, clades with identical branching orders in both phylogenies were collapsed (Fig. 1A). Despite the high branch support, substantial topological conflicts were observed between the two phylogenies, suggesting that the observed mito-nuclear phylogenetic discordance are not artifacts of insufficient phylogenetic resolution. The topological difference (that is, normalized Robinson–Foulds distance) between the two trees was 0.306, indicating a considerable level of topological discordance. In addition, when topological differences were examined for each of the five major insect orders, we found that the degree of mito-nuclear phylogenetic discordance varied substantially (Fig. 1C), with Lepidoptera exhibiting the greatest incongruence (nRF distance = 0.446) and Hymenoptera the lowest incongruence (nRF distance = 0.158). These findings suggest that the mito-nuclear phylogenetic discordance is widespread across insects and cannot be explained by insufficient phylogenetic signals.

In addition, we examined whether model choice in sequence evolution could explain the observed topological differences. To assess this, mitochondrial phylogenies were reconstructed from the concatenated amino acid sequences of 13 mitochondrial genes across 472 species via a site-heterogeneous mixture model (mtInv + F + G4 + C60). As a result, we found that the site-heterogeneous mixture model did not eliminate the mito-nuclear phylogenetic discordance (Supplementary Fig. 1), suggesting that the introduction of the complex model also cannot explain the mito-nuclear phylogenetic discordance detected in our dataset.

### Impact of nine gene properties on the mito-nuclear phylogenetic discordance

**Fig. 2:**
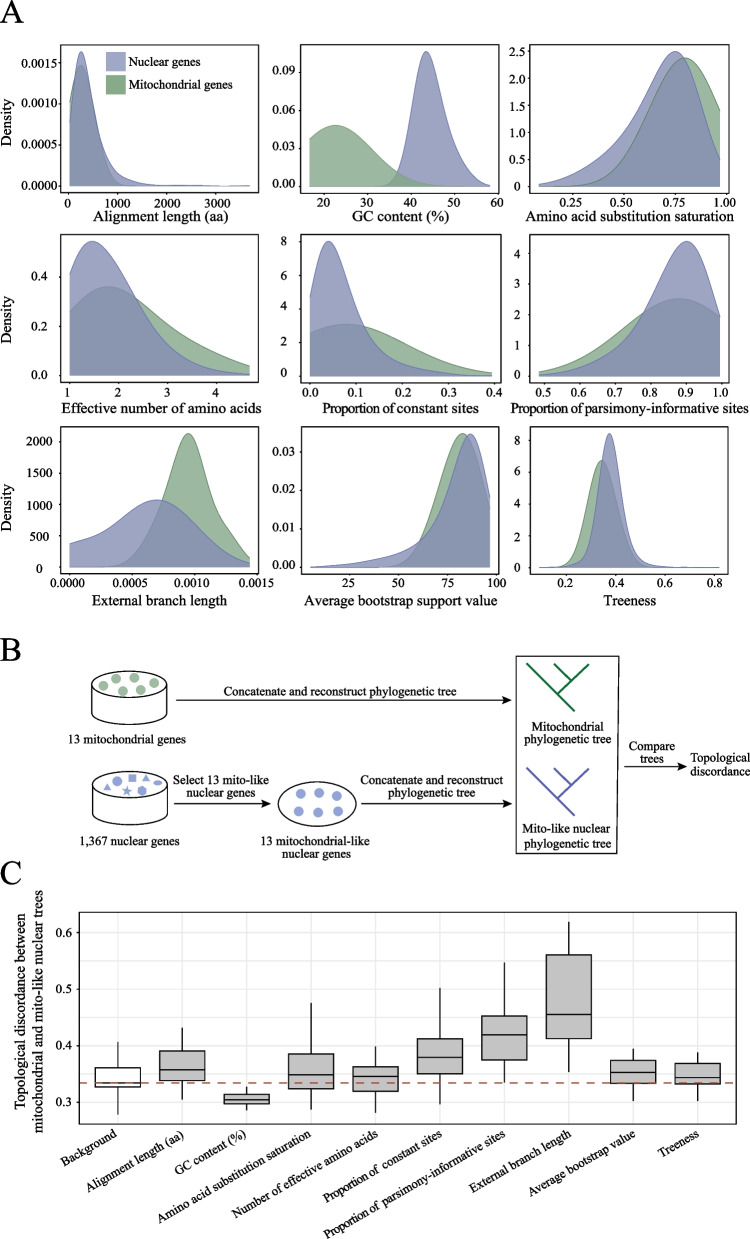
Impact of gene properties on the discordance between mitochondrial and nuclear phylogenies. **A** Comparison of nine gene properties between nuclear and mitochondrial genes, including alignment length, GC content, amino acid substitution saturation, effective number of amino acids, proportion of constant sites, proportion of parsimony-informative sites, external branch length, average bootstrap support value, and treeness. The green and blue colors represent mitochondrial and nuclear genes, respectively. **B** Workflow of the property-matching strategy for assessing the impact of gene properties on mito-nuclear phylogenetic discordance. Color green represents 13 mitochondrial protein-coding genes, whereas blue represents nuclear genes. The phylogeny inferred from 13 mitochondrial protein-coding genes served as the reference tree. For a given gene property, we selected 13 mito-like nuclear genes whose values matched those of 13 mitochondrial genes (see Methods for details). Each selection and tree reconstruction procedure was repeated 20 times. In addition, we randomly selected 13 mito-like nuclear genes as the background. **C** Effect of the property-matching strategy on the basis of nine gene properties. The red dashed line indicates the median topological discordance of the background group. Each boxplot shows the distribution of topological discordance between the mito-like phylogenetic tree and the mitochondrial phylogenetic tree

We investigated whether gene properties underlie the topological differences between the mitochondrial and nuclear phylogenies. We systematically quantified nine gene properties, including alignment length, GC content, amino acid substitution saturation, effective number of amino acids, proportion of constant sites, proportion of parsimony-informative sites, external branch length, average bootstrap support value, and treeness (proportion of internal branch lengths over all branch lengths) (Fig. 2A and Supplementary Table 1). Among these nine gene properties, we found that the distribution of the GC content of mitochondrial genes was substantially different from the GC content of nuclear genes, and most mitochondrial genes presented a lower GC content than nuclear genes did.

Next, we implemented a property-matching strategy (Fig. 2B), in which 13 nuclear genes were selected to match the 13 mitochondrial genes on the basis of similarity in a specific gene property, and we examined whether sampling mito-like nuclear genes would make them topologically similar to the mitochondrial phylogeny. In brief, for a given property, we sampled a set of 13 mito-like nuclear genes whose properties are similar to those of the 13 mitochondrial genes. We subsequently compared the ML tree inferred from the 13 concatenated mito-like nuclear genes with the phylogeny inferred from the 13 concatenated mitochondrial genes. We also created a background that randomly selected 13 nuclear genes. This property-matching strategy was conducted 20 times for each gene property. We found that the background presented a median nRF value of 0.334 between 13 mitochondrial genes and 13 random nuclear genes. Compared with the background, among all nine properties, only the GC content had the ability to reduce the phylogenetic discordance between 13 mitochondrial genes and 13 mito-like nuclear genes, whereas the remaining properties did not.

To further explore these findings, we compared the GC content at the first, second, and third codon positions (GC1, GC2, and GC3, respectively) between the mitochondrial and nuclear genes. We found that codons in mitochondrial genes exhibited a remarkable bias against codons ending in G or C, indicating a strong preference for A/T-ending codons, whereas codons in nuclear genes exhibited a more balanced pattern between the first, second, and third codon positions (Supplementary Fig.  2 A). Since GC content bias can directly affect amino acid composition, we next examined the amino acid usage frequencies of the two genomes. We found that the mitochondrial genome favored amino acids encoded by low-GC codons (e.g., Leu, Ile, Phe, Met, Asn, and Tyr), whereas the nuclear genome favored amino acids encoded by high-GC codons (e.g., Ala, Arg, Glu, and Asp) (Supplementary Fig. 2B). Taken together, these results demonstrate that differences in GC content play important roles in the observed mito-nuclear phylogenetic discordance.

### Phylogenetic discordance associated with the content of GC within nuclear genes

**Fig. 3:**
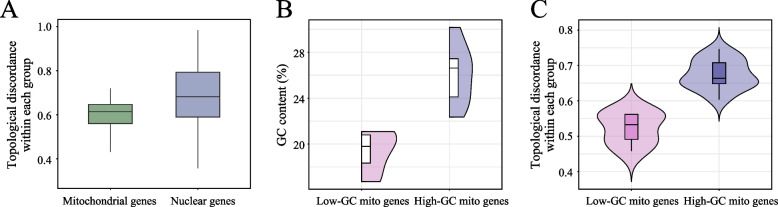
GC content contributes to topological discordance among mitochondrial genes. **A** Comparison of pairwise topological discordance among mitochondrial genes and nuclear genes. **B** Distribution of the GC contents of the two mitochondrial gene groups. **C** Distribution of topological discordances among each of the two mitochondrial gene groups

The above analyses focused primarily on investigating the discordance between mitochondrial and nuclear genes. However, the understanding of the phylogenetic discordance within mitochondrial genes or nuclear genes themselves is limited. To do so, we first calculated pairwise topological differences among the mitochondrial genes and among the nuclear genes. Interestingly, we found that mitochondrial genes presented greater topological similarity to each other than did nuclear genes (Fig. 3A). As mitochondrial genes generally have lower GC contents than nuclear genes do, we hypothesized that low-GC content genes present a greater level of phylogenetic similarity to each other than do high-GC content genes.

**Fig. 4:**
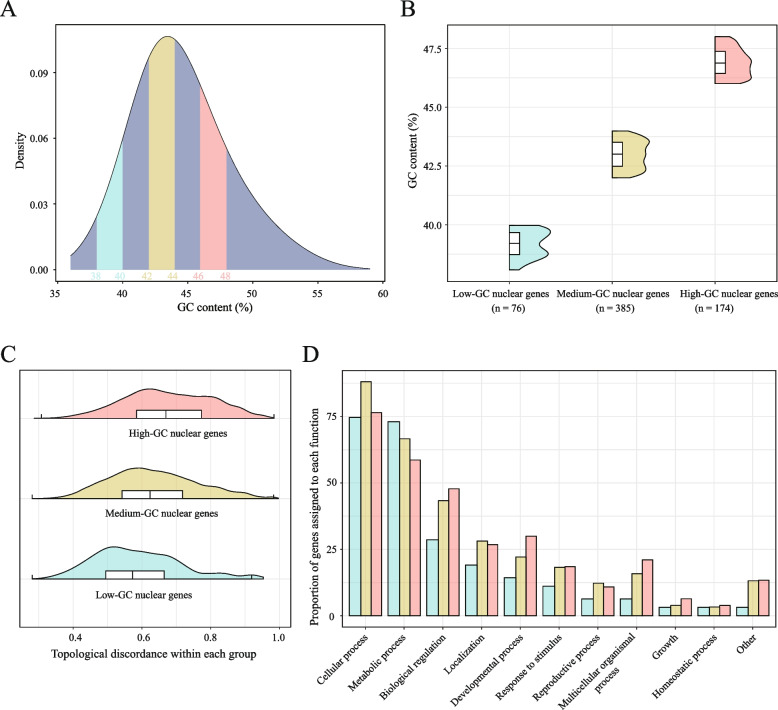
GC content contributes to topological discordance among nuclear genes. **A** Distribution of GC contents in nuclear genes, highlighting three non-overlapping groups. The GC content thresholds for each group are labeled above the x-axis in corresponding colors. **B** Distribution of the GC content for each nuclear gene group. The number of genes in each group is shown below the x-axis. **C** Distribution of pairwise topological discordance among each nuclear gene group. **D** Proportion of genes associated with different Gene Ontology (GO) terms in each gene group. The bars represent the proportion of genes annotated with each GO term relative to the total number of genes within each group

To validate our hypothesis, we divided the mitochondrial genes into two groups that did not overlap in terms of GC content (Fig. 3B). When pairwise topological differences among each group were examined, we found that low-GC content mito-genes were more topologically similar to each other than high-GC content mito-genes were (Fig. 3C). Similarly, we also divided the 1,367 nuclear genes into three groups on the basis of their GC content: the low-GC group (38–40%), the medium-GC group (42–44%), and the high-GC group (46–48%) (Fig. 4A, 4B). Notably, the range of the GC content in each group was 2%, suggesting that the degree of variation in the GC content in each group was similar. Pairwise topological differences (that is, nRF distances) were then calculated among gene trees within each group. Within-group comparisons revealed a clear trend of increasing topological differences with increasing GC content (Fig. 4C), indicating that low-GC nuclear genes are more topologically similar to each other.

Finally, we investigated whether differences in GC content also reflect underlying functional differentiation among nuclear genes. Gene Ontology (GO) analysis, in which detailed GO terms were consolidated into broader biological process categories, revealed clear patterns across the three GC groups (Fig. 4D). The high-GC nuclear genes represented the greatest proportion of genes involved in several complex biological processes: biological regulation, developmental process, response to stimulus, multicellular organismal process, growth, and homeostatic process. However, the low-GC nuclear genes represented the greatest proportion of genes involved in metabolic processes. Together, these results indicate that the GC content not only influences the phylogenetic tree but also reflects functional differentiation within the nuclear genome.

## Discussion

The phylogenetic trees inferred from mitochondrial and nuclear genomes often exhibit conflicting topologies, yet the underlying causes of this discordance remain unclear [25–27]. In this study, we systematically investigated the mito-nuclear discordance among 472 insect species by quantifying nine gene properties. Our analyses revealed that the GC content influences not only phylogenetic discordance but also underlying functional differentiation.

As the most commonly sampled markers for many groups [28–30], mitochondrial genomes are consistently shorter than are nuclear genomes, and differences in gene alignment length are generally considered to contribute to the mito-nuclear phylogenetic discordance. However, our analyses revealed that mito-nuclear phylogenetic discordance was not reduced when using nuclear genes that are similar to mitochondrial genes in alignment length, suggesting that gene alignment length does not explain the observed conflicts. In addition, mitochondrial genes are generally maternally inherited, leading them to share a similar evolutionary history. However, when we compared mitochondrial genes grouped by GC content, substantial topological discordances were still observed between low-GC and high-GC mitochondrial genes. In addition to biological factors, technical issues have also been proposed as contributors to mito–nuclear phylogenetic discordance. One commonly cited example is the misapplication of sequence evolution models, especially in cases involving strong compositional heterogeneity or substitutional saturation [31, 32]. Because mitochondrial genomes typically evolve faster than nuclear genomes do, applying more complex models is often expected to improve phylogenetic inference. However, in our study, we found that increasing the evolutionary model complexity of mitochondrial genes had a minimal impact on reducing mito-nuclear phylogenetic discordance [33–35]. These results indicate that the gene alignment length, inheritance pattern, and evolutionary model complexity might not explain the observed mito-nuclear phylogenetic discordance.

After examining nine gene properties, we identified the GC content as the gene property most strongly associated with the mito-nuclear phylogenetic discordance. In addition, within mitochondrial genomes or nuclear genomes, genes with lower GC contents produced more topologically consistent trees than those with higher GC contents. One plausible explanation is that GC-rich genomic regions experience higher recombination rates driven by GC-biased gene conversion, which might lead to greater topological discordance [36–38]. Additionally, the GC content is known to be correlated with the gene expression and biological function of genes [22, 39–41]. Consistent with these observations, we found that low-GC nuclear genes exhibit different biological functions compared with high-GC nuclear genes.

Our results bridge molecular composition and evolutionary inference, suggesting that nucleotide compositional heterogeneity, particularly GC content, can influence not only phylogenetic reconstruction but also function. Nevertheless, several limitations should be acknowledged. Multiple biological and analytical processes have been proposed to explain nuclear phylogenomic incongruence, including incomplete lineage sorting, introgression, hybridization, sex-biased dispersal, and horizontal gene transfer [26, 27, 35, 42–48]. Future research integrating those factors will be essential to test whether they contribute to the mito-nuclear phylogenetic discordance.

## Materials and methods

### Data acquisition

We utilized the mitochondrial and nuclear genome data of 472 insects from a recent study [24], which included 13 protein-coding mitochondrial genes (PCGs) and 1,367 single-copy nuclear genes.

### Phylogenetic tree construction

To infer genome-scale phylogenetic relationships among 472 insects, we introduced 15 outgroup species from *Entognatha*. We used two datasets to infer the phylogenetic tree: (1) amino acid sequences of 13 mitochondrial protein-coding genes and (2) amino acid sequences of 1,367 nuclear genes. For each gene, the sequences were aligned via the program MAFFT version 7.299b [49] with the options ‘–auto–maxiterate 1000’, and the alignment was trimmed via trimAl version 1.4.rev15 [50] with the options ‘-gappyout-colnumbering’. For each dataset, trimmed alignments were concatenated into a supermatrix via PhyKit [51]. Concatenation-based maximum likelihood (ML) trees were constructed via IQ-TREE multicore version 2.1.4 [52] with 1,000 ultrafast bootstrap replicates to assess branch support. We used the best-fitting model LG + G4 to infer the phylogenetic tree from the nuclear dataset and the best-fitting model mtInv + F + G4 for the mitochondrial dataset. Phylogenetic trees were visualized via iTOL version 7.2 [53].

In addition to the concatenation-based trees, we also constructed individual gene trees for each dataset. For the mitochondrial genome, individual maximum-likelihood (ML) gene trees were inferred via IQ-TREE (multicore version 2.1.4) with “iqtree –runs 10 -st AA -s [alignment_file] -m mtInv + F + G4 -bb 1000 -pre [tree_name]”. For the nuclear genome, individual ML gene trees were inferred via “iqtree –runs 10 -st AA -msub nuclear -s [alignment_file] -m TEST -bb 1000 -pre [tree_name]”.

To further assess the influence of alternative sequence evolution models on the mitochondrial phylogenetic tree, we used an additional site-heterogeneous model, C60, with the option ‘-m mtInv + F + G4 + C60’.

### Phylogenetic tree comparison

Topological discordance between phylogenetic trees was measured by the normalized Robinson-Foulds (nRF) distance [54]. The nRF distance ranges from 0 to 1, where lower values denote greater topological similarity between trees. We calculated the nRF distance with the ETE3 package version 3.1.3 [55].

### Gene properties

We evaluated nine genomic properties for both the nuclear and the mitochondrial genomes. For each gene alignment, we calculated the following: (a) Alignment length: the total number of amino acid sites. (b) GC content: percentage of guanine and cytosine nucleotides. (c) Amino acid substitution saturation estimated via PhyKit [51] measures the extent of substitutions in a multiple sequence alignment, ranging from 0 (highly saturated) to 1 (minimally saturated). (d) The effective number of amino acids: a metric of per-site compositional diversity, as defined by Szánthó [56], ranging from 1.0 (a single amino acid exclusively used) to 20.0 (all amino acids uniformly distributed). (e) Proportion of constant sites and (f) Proportion of parsimony-informative sites extracted from the IQ-TREE log files. (g) External branch length: the median external branch length in an ML gene tree. (h) Average bootstrap support value: the mean bootstrap support value across all nodes. (i) Treeness: ratio of internal branch length to total branch length, defined by Phillips and Penny [57]. These metrics provide insights into the overall tree shape, support, and proportion of phylogenetic signals distributed within internal branches.

### Property-matching strategy for selecting mitochondrial-like nuclear genes

To investigate the role of nine gene properties in the mito-nuclear phylogenetic discordance, we implemented a property-matching strategy that identifies mito-like nuclear genes and evaluates their phylogenetic similarity to mitochondrial trees (Fig. 2B). A reference mitochondrial phylogeny was inferred from a concatenated supermatrix of amino acid alignments comprising 13 protein-coding mitochondrial genes. The tree was inferred via IQ-TREE v2.2.0 via mtInv + F + G4 with 1,000 ultrafast bootstrap replicates to assess the node support value. For each gene property, 13 nuclear genes whose values matched those of the 13 mitochondrial genes were chosen. For example, in the alignment length test, a nuclear gene was considered similar to a mitochondrial gene if its alignment length differed by less than ± 5% from that of the mitochondrial gene. These 13 mito-like nuclear genes were concatenated into a supermatrix and used to construct a phylogenetic tree with IQ-TREE v2.2.0. This sampling and tree-building procedure was repeated 20 times. The other test groups were subjected to the same procedures.

### Functional annotation analysis

For genes in the low-, medium-, and high-GC groups, we collected the Gene Ontology (GO) terms for each gene and compiled them into 18 Biological Process (BP) categories via the Python package goatools [58].

## Supplementary Information

Supplementary Material 1. Figure S1 Nuclear and mitochondrial phylogenies inferred under different evolutionary models. The nuclear phylogeny (top) was inferred via the LG+G4 model, whereas the mitochondrial phylogenies (bottom left and right) were reconstructed via the mtInv+F+G4 and mtInv+F+G4+C60 models, respectively. Topological discordances (nRF) between each pair of trees are shown along the connecting lines. Branch colors correspond to different insect orders, similar to those in Fig. 1A. Figure S2 Nucleotide preferences at different codon positions correlate with amino acid usage in nuclear and mitochondrial genes. (A) Distribution of the GC content at the first (GC1), second (GC2), and third (GC3) codon positions for the mitochondrial (green) and nuclear (blue) genes. (B) Proportion of twenty standard amino acids in the mitochondrial (green) and nuclear (blue) genes. Note that the difference between nuclear and mitochondrial codon codes was considered.

Supplementary Material 2. Table S1 Detailed data on nine gene properties of each mitochondrial and nuclear gene.

## Data Availability

All data and materials for this study are available upon request from the corresponding author (xingxingshen@zju.edu.cn).
