# Supplementary material for: Dissecting discordance of mitochondrial and nuclear phylogenetic trees in insects
Source: Crop Health. 2025 Dec 12;3(1):23. doi: 10.1007/s44297-025-00062-3 (PMC12825919; doi:10.1007/s44297-025-00062-3)

Supplementary Figure 1

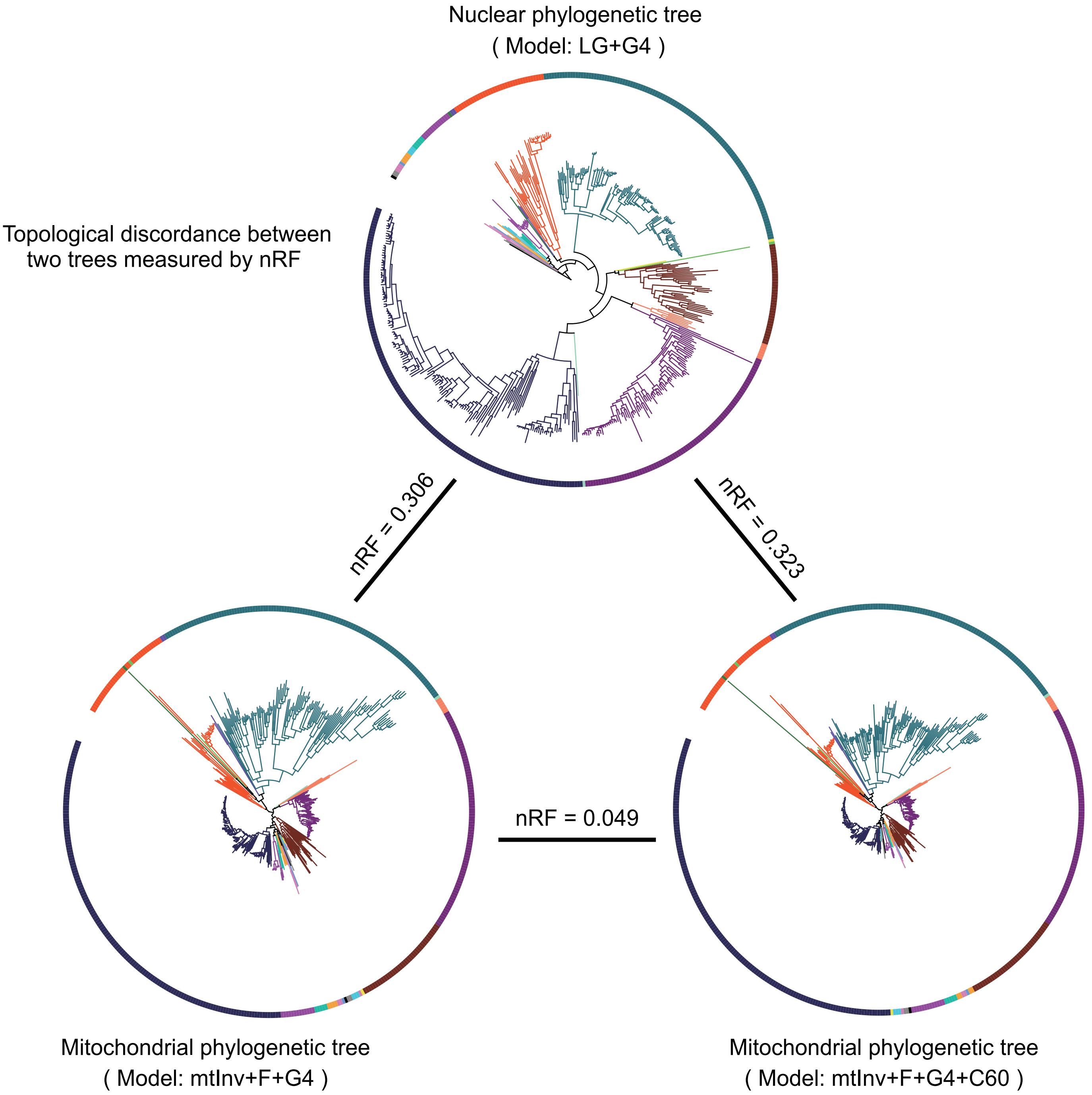

Supplementary Figure 2

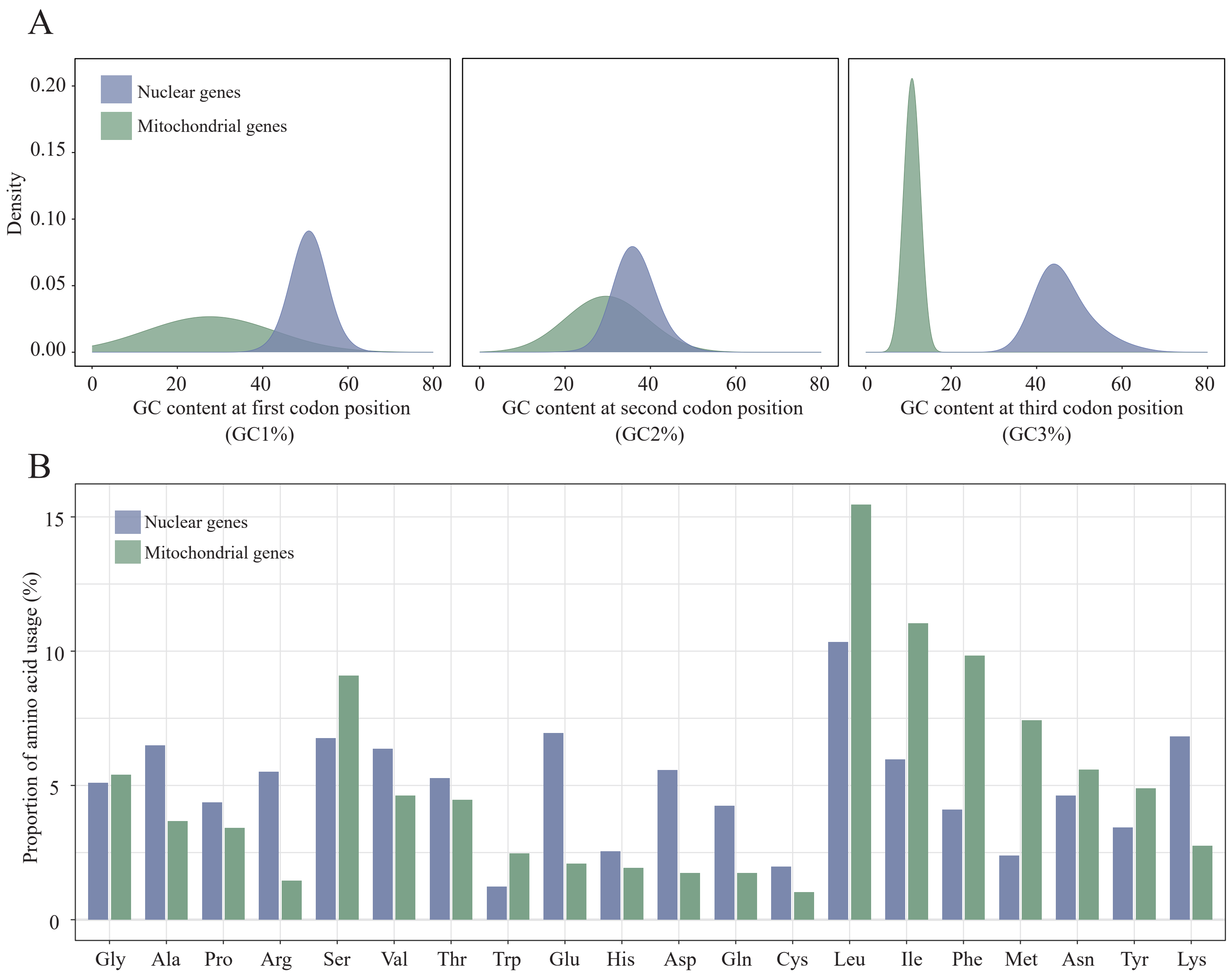

Supplement: Supplementary file 1 — Supplementary Material 1. Figure S1 Nuclear and mitochondrial phylogenies inferred under different evolutionary models. The nuclear phylogeny (top) was inferred via the LG+G4 model, whereas the mitochondrial phylogenies (bottom left and right) were reconstructed via the mtInv+F+G4 and mtInv+F+G4+C60 models, respectively. Topological discordances (nRF) between each pair of trees are shown along the connecting lines. Branch colors correspond to different insect orders, similar to those in Fig. 1A. Figure S2 Nucleotide preferences at different codon positions correlate with amino acid usage in nuclear and mitochondrial genes. (A) Distribution of the GC content at the first (GC1), second (GC2), and third (GC3) codon positions for the mitochondrial (green) and nuclear (blue) genes. (B) Proportion of twenty standard amino acids in the mitochondrial (green) and nuclear (blue) genes. Note that the difference between nuclear and mitochondrial codon codes was considered. [file 44297_2025_62_MOESM1_ESM.pdf]
